# Temporal dynamics of bacteria-plasmid coevolution under antibiotic selection

**DOI:** 10.1038/s41396-018-0276-9

**Published:** 2018-09-12

**Authors:** Michael J. Bottery, A. Jamie Wood, Michael A. Brockhurst

**Affiliations:** 1 Department of Biology, University of York, Wentworth Way, York YO10 5DD UK; 2 Department of Mathematics, University of York, Heslington, York YO10 5DD UK; 3 Department of Animal and Plant Sciences, University of Sheffield, Western Bank, Sheffield, S10 2NT UK

**Keywords:** Antibiotics, Molecular evolution

## Abstract

Horizontally acquired genes can be costly to express even if they encode useful traits, such as antibiotic resistance. We previously showed that when selected with tetracycline, *Escherichia coli* carrying the tetracycline-resistance plasmid RK2 evolved mutations on both replicons that together provided increased tetracycline resistance at reduced cost. Here we investigate the temporal dynamics of this intragenomic coevolution. Using genome sequencing we show that the order of adaptive mutations was highly repeatable across three independently evolving populations. Each population first gained a chromosomal mutation in *ompF* which shortened lag phase and increased tetracycline resistance. This was followed by mutations impairing the plasmid-encoded tetracycline efflux pump, and finally, additional resistance-associated chromosomal mutations. Thus, reducing the cost of the horizontally acquired tetracycline resistance was contingent on first evolving a degree of chromosomally encoded resistance. We conclude therefore that the trajectory of bacteria-plasmid coevolution was constrained to a single repeatable path.

## Main

The acquisition of mobile genetic elements (MGEs) encoding ecologically important functions promotes evolutionary innovation in bacteria [1]. The rapid spread of antibiotic resistance among bacterial pathogens is facilitated by the horizontal gene transfer (HGT) of multidrug resistance (MDR) plasmids [2, 3]. However, MDR plasmid acquisition is often costly for the host cell leading to conflict between the chromosome and the plasmid [4, 5]. These conflicts, which may arise through disruption of host cellular networks or cytotoxicity of plasmid-encoded proteins [6], can be ameliorated through compensatory evolution to reduce the cost of plasmid carriage, and, in some cases, reciprocal co-adaptation of the chromosomal and plasmid replicons [7, 8]. We previously showed that when selected with tetracycline, *Escherichia coli* carrying the tetracycline-resistance plasmid RK2 evolved mutations on both replicons that together provided increased tetracycline resistance at reduced cost [7]. In the previous study, we confirmed for a single population that the emergence of chromosomal resistance preceded mutation of the plasmid, but were unable to test the repeatability of this coevolutionary trajectory between multiple independent populations. Here we investigate the temporal dynamics of intragenomic coevolution by whole-genome sequencing 27 additional evolved clones isolated through time from three independently evolving populations selected with tetracycline.

**Fig. 1:**
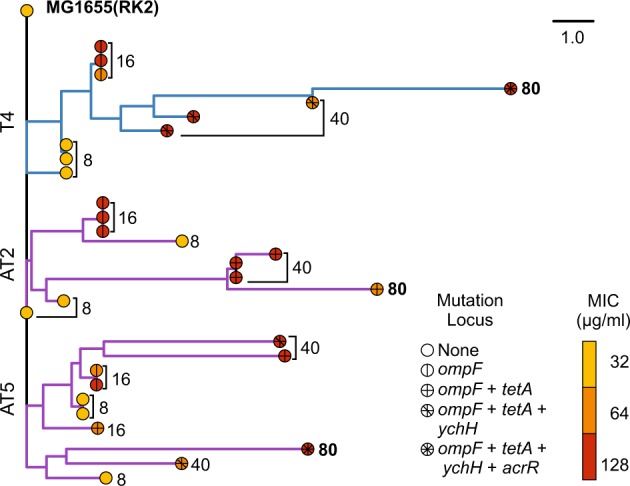
Phylogeny of sequenced clones isolated from populations T4, AT2, and AT5 rooted using the ancestral MG1655(RK2). The distance matrix used to produce the tree was constructed from the binary presence or absence of mutations, specific to the nucleotide level, relative to the ancestral strain. The scale bar represents number of mutations. Branch tips are coloured by the MIC of the sequenced clone, tip symbols represent the presence of parallel mutations within the evolved clone. Tips are labelled with the transfer from which the clone was isolated from. Blue branches show the lineage of clone evolved within the tetracycline only treatment, purple branches show two independent populations evolved under tetracycline plus ampicillin treatment. MIC curves for each transfer are presented in Fig. S3. Full genotypes of sequenced strains are available in Table S1

Three clones from transfers 8, 16, and 40 from three independent populations under tetracycline treatment were newly sequenced. These together with the previously published genomes from transfer 80 [7] were analysed to assess the order of mutations within each population. Construction of phylogenies based on the presence or absence of mutations shows that there was considerable within-population diversity across all three populations (Fig. 1, for Methods see Supplementary Information). The accumulation of short branches at the base of the trees suggests divergence during the early stages of evolution. Subsequently, a single lineage appears to dominate population T4, arising early within the experiment, suggestive of the stepwise acquisition of beneficial mutations with subsequent selective sweeps within this lineage. Likewise, in population AT2 a single lineage dominated by transfer 16 but was superseded by a second lineage that appears to have diverged earlier in time. Whereas, in population AT5 the phylogeny has deeper branching, indicating that different mutations emerged in parallel and lineages coexisted for a longer duration than observed in the T4 or AT2.

**Fig. 2:**
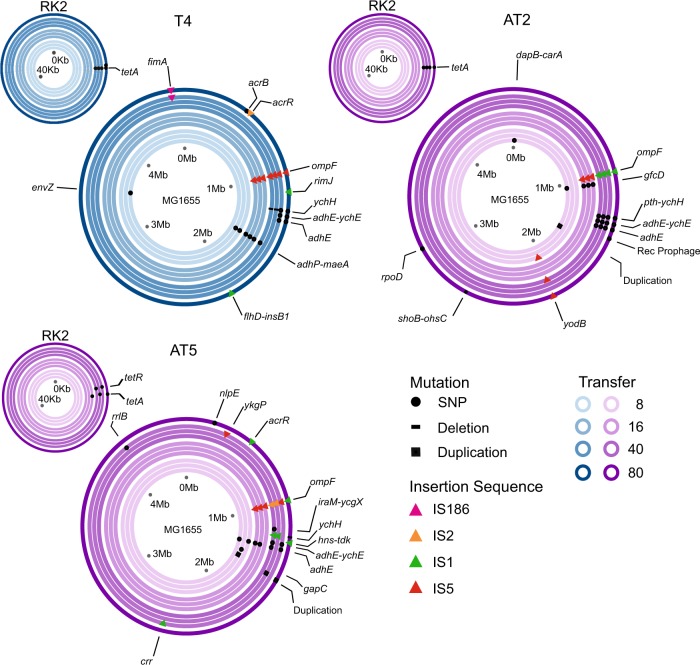
Genomic changes observed at transfers 8, 16, 40, and 80 within populations under tetracycline treatment. Concentric rings represent *E. coli* chromosomes or RK2 plasmids, ring colour represent the time point the clones were isolated (see keys); inner lighter rings to outer darker rings represent progression through time. Sets of three concentric rings are representative of three independent clones isolated from the same time point within the populations. Points on the plot represent mutations at specific loci, circles = non-synonymous mutations, bars = deletions, squares = duplications, and triangles = IS elements (colours show type of IS element, see key). Genome sequences from transfer 80 are previously published in ref. [7]

In the original study a single clone was sequenced from the endpoint transfer (transfer 80) from each population. Adaptive mutations were identified as those showing parallel evolution in multiple replicate populations within tetracycline treatments, which is strong evidence that mutations at these loci were the targets of natural selection [7]. Hereafter we focus our analysis on this subset of mutated loci. Across all three populations, the order in which these parallel mutations were acquired was shared. The first parallel mutation gained was the loss of function of the outer membrane porin *ompF* via the acquisition of an IS element at transfer 16 (Fig. 2). Many classes of antibiotics, including tetracycline, cross the outer membrane of Gram-negative bacteria via outer membrane porin proteins, including OmpF [9, 10]. Loss of function of the *ompF* gene reduces membrane permeability and is associated with resistance phenotypes in clinical isolates of *E. coli* [11]. Clones isolated from transfer 16 had significantly increased resistance to tetracycline when compared to those from transfer 8 or the ancestral strain (Fig. 1 and Fig. S3, Post hoc Tukey Tests: Anc(RK2):Transfer 16 all *p* < 0.05, see Table S2 for ANOVA tables), and this was significantly associated with mutation of *ompF* (Pearson Chi-squared, *χ*^2^ = 30, *p* < 0.01, Bonferroni corrected). By transfer 16 all clones also had a significantly reduced lag phase compared to the ancestral strain (Fig. S4, Post hoc Tukey Tests: Anc(RK2):Transfer 16 all *p* < 0.05, see Table S3 for ANOVA tables). IS elements were observed within all subsequent time points, however, only population T4 maintained the same IS element within *ompF* throughout the experiment. Populations AT2 and AT5 had multiple different IS elements within the *ompF* loci (Fig. 2). These results suggest that the loss of function of OmpF could evolve independently multiple times within a single population. Similar MIC values among these independent lineages suggest that these mutations had equivalent functional consequences (Fig. S3).

Non-synonymous single nucleotide polymorphisms (SNP) within the plasmid-encoded *tetA* or *tetR* genes, the tetracycline specific efflux pump and its negative regulator genes, respectively, [12] were the next parallel mutations observed (Fig. 2). These mutations reduce the cost of the plasmid, but cause weaker resistance to tetracycline, in the ancestral MG1665 [7]. There was no significant difference in the MIC between clones from transfer 16 and 40 (Post hoc Tukey Tests: Transfer 16:Transfer 40 *p* > 0.05), suggesting that *ompF* mutations compensated for the reduced resistance provided by the mutated tetracycline efflux pump [7].

Mutations in the hypothetical general stress response gene, *ychH* [13], were the next parallel mutations observed (Fig. 2) followed by mutation of *acrR*, encoding the negative regulator of the *acrAB* MDR cassette [14]. Mutation of *acrR* causes overexpression of the AcrAB-TolC multidrug efflux pump, leading to MDR phenotypes [15, 16]. Genetically different mutations, including premature stop codons and acquisition of IS elements within *ychH* and *acrR*, occurred within multiple lineages of the same population (Fig. 2). However, the acquisition of these secondary resistance-associated mutations was not associated with resistance (Pearson Chi-squared, *ychH χ*^2^ = 3.91, *p* > 0.05, *acrR χ*^2^ = 2.14, *p* > 0.05, Bonferroni corrected), providing no increase in tetracycline MIC (Post hoc Tukey Tests, populations T4 and AT5: Transfer 16:Transfer 40:Transfer 80 all *p* > 0.05; Fig S3). In contrast, the endpoint clone isolated from the AT2 population lacked mutations in either gene but showed reduced tetracycline resistance (Fig. S3, not significantly different from ancestral MIC, Anc(RK2):AT2 Transfer 80 *p* = 0.481).

We have shown that chromosome-plasmid coevolution under antibiotic selection was highly repeatable between three independently evolving populations. Similarly, within these populations, multiple lineages arose which acquired genetically distinct but functionally equivalent mutations in the same order, further emphasising how strikingly repeatable the observed bacteria-plasmid coevolution was. Mutations in a chromosomally encoded outer membrane porin increasing tetracycline resistance and reducing lag phase were followed by mutations impairing the costly plasmid-encoded tetracycline efflux pump [17, 18], and subsequent mutation of chromosomally encoded stress response and multidrug efflux systems. The phenotypic effects upon resistance and growth potentially govern the repeatable order of mutations, suggesting that reducing the cost of expressing the horizontally acquired tetracycline resistance was contingent on first evolving a degree of chromosomally encoded resistance. The earliest mutation, *ompF*, had the greatest phenotypic effect on both tetracycline resistance and growth possibly suggesting a role for diminishing returns epistasis in the order that mutations were selected [19]. The fitness landscape of antibiotic resistance mutations can constrain the order in which resistance mutations are acquired [20], giving rise in both experimental [21] and clinical [22, 23] studies to strikingly predictable trajectories of antibiotic resistance evolution. Here we show that the trajectory chromosome-plasmid coevolution under antibiotic selection is similarly highly predictable, which may contribute to our understanding of successful pathogenic clades following their acquisition of MDR plasmids [24, 25]. Our data highlight the importance of coevolution for the continued evolutionary adaptation of resistant strains under antibiotic selection.

## Electronic supplementary material

Supplementary information

Supplementary Table 1
