# Supplementary material for: Temporal dynamics of bacteria-plasmid coevolution under antibiotic selection
Source: ISME J. 2018 Sep 12;13(2):559–62. doi: 10.1038/s41396-018-0276-9 (PMC6330079; doi:10.1038/s41396-018-0276-9)
Supplement: Supplementary file 1 — Supplementary information [file 41396_2018_276_MOESM1_ESM.docx]

**Supplementary information for “Temporal dynamics of bacteria-plasmid coevolution under antibiotic selection”**

This document contains:

Supplementary Methods

Supplementary Figures 1-4

Supplementary Tables 2, 3

Supplementary References

**Supplementary Methods**

*Experimental Design*

The evolution experiment, described previously [1], coevolved *Escherichia coli* MG1655 with the multidrug resistance plasmid RK2 in various antibiotic treatments, to which the RK2 plasmid provided resistance. Cultures were grown at 37°C in 50 ml microcosms containing 5 ml of Oxoid® Nutrient Broth (NB) shaken at 180 rpm. Six independent biological replicates (populations) were initiated for each treatment, consistent with previous evolution experiments and sufficient to detect treatment specific parallel evolution [2]. Constant tetracycline treatments were supplemented with 10 μg ml^-1^ tetracycline (populations T1-6) or 100 μg ml^-1^ ampicillin plus 10 μg ml^-1^ tetracycline (populations AT1-6). The populations were evolved for ~530 generations (80 days), through daily transfers of 50 μl of saturated culture into 5 ml of fresh media supplemented with antibiotics specific to the treatment, yielding roughly 6.6 generations per day. OD_600_ of populations were measured daily. 500 μl samples from each population were frozen at -80°C on day zero and every 8 days (~53 generations) thereafter in 25% glycerol. Whole populations were also diluted and spread onto nutrient agar every 8 days, with 20 individual colonies selected at random and frozen at -80°C. A single clone from day 80 (generation 530) the end of the experiment from each population was randomly selected for sequencing, along with 3 clones from transfers 8, 16 and 40 in populations T3, AT2 and AT5. Populations under constant tetracycline treatment were selected for further analysis based on the clones’ final sequenced genotype, so that the population contained insertion sequence (IS) elements within *ompF* and did not exhibit a hypermutator phenotype (no mutations within *mutL* of *mutS*). The time points chosen for sequencing were selected to provide samples prior to the observation of IS elements within the population based on whole population PCR (Fig. S1), as well as the time point at which they were first observed and 24 transfers after first observing IS elements within the population.

*PCR tracking of mutations*

The frequency of IS mutation of the *ompF* genes was tracked within populations T4, AT2 and AT5 though whole population PCR. PCR primers amplifying *ompF* (Fw: ACTTCAGACCAGTAGCCCAC, Rv: GCGCAATATTCTGGCAGTGA) identified IS elements; a PCR short product of 716 bp indicated no insertion sequence, a product of 1484 bp indicted IS1 and a long product of 1911 bp indicted IS5 within *ompF*. 5 μl of frozen whole populations from every 8 transfers was mixed with 10 μl of nuclease free water, 1 μl of which was used as template for the PCR, resulting in approximately 1 × 10^5^ cells per PCR reaction. IS elements were never observed within the control population which coevolved with the RK2 plasmid in the absence of antibiotic treatment (N1). Standard PCR reactions were performed using Go Taq Green Master Mix (Promega) 0.4 μM of each primer, on a program of 95°C for 5 min, 30 cycles of 95°C for 30 s, 60°C for 30 s, and 72°C for 2min, with a final extension of 72°C for 5 mins.

*Sequencing and analysis*

Three clones without IS elements in *ompF* were randomly selected from transfer 8 from populations T4, AT2, AT5 for sequencing. Three random clones containing IS elements within *ompF* from populations T4, AT2, AT5, transfers 16 and 40 were also selected for sequencing. Each clone was grown for 24 hours until saturation in the same selective environment as they evolved in, before total genomic extraction using the DNeasy Blood and Tissue extraction kit (Qiagen) according to the manufactures instructions. The integrity of the DNA was assessed on a 0.75% agarose gel, and concentration estimated by Qubit dsDNA BR Assay Kit (Thermo Fisher Scientific). Total genomic DNA was sequenced by MicrobesNG (http://www.microbesng.uk), which is supported by the BBSRC (grant number BB/L024209/1), using 2 × 250 base pair paired-end reads on the Illumnia MiSeq platform. Sequence adapters were trimmed using Trimmomatic [3] and quality assessed using Samtools [4], BedTools [5] and bwa-mem [6] by MicrobesNG. Trimmed reads were mapped to the *E. coli* MG1655 K-12 and RK2 plasmid genomes available on NCBI, accession numbers U00096.3 and BN000925.1, respectively, using the Burrows-Wheeler short read aligner [6]. Variants were called using GATK’s Unified Genotyper [7] and annotated using SnpEff [8]. Variants were filtered on base quality, coverage and single nucleotide polymorphisms (SNPs) around gaps. Insertion sequences were detected based on an over representation of larger inner mate gaps between mapped paired reads and structural variants were detected using Breakdancer [9]. All variants were confirmed using the Integrated Genome Viewer [10]. To produce a set on mutations accumulated throughout the evolution experiment, variants that were also present in the previously sequenced ancestral strain [1] were excluded. In total we observed 112 mutations across 30 clones (Fig. S2/S3), comprising of: 60 point mutations, 40 IS elements, 8 deletions and 4 duplications. Of these mutations 24 were intergenic variants and 3 were synonymous substitutions.

*Phylogenetic analysis*

The phylogenetic trees were produced based on the binary presence and absence of all observed mutations within the data set for each sequenced clone. This gives each observed mutation equal weight when building the trees, which is appropriate when variants are under strong selection [11]. Trees were built using the neighbour joining method provided by the R package *ape* {ape 4.0} [12] and were rooted to no observed variants, i.e. the ancestral strain harbouring RK2. The strong parallelism of mutations between populations resulted in clones from different populations clustering within the same clades when a composite tree was produced. However, as the populations evolved independently, separate trees were produced for each population and combined into a single plot.

*Minimum inhibitory concentrations*

To measure the minimum inhibitory concentration (MIC) of tetracycline, clones were grown over night in 5 ml of NB. The saturated cultures were sub-cultured 50 μl into 5 ml of fresh NB and allowed to grow until an OD_600_ of 0.5. These sub-cultures were then diluted 1:400, to an initial density of ~5×10^5^ CFU/ml, in 96-well plates containing a log^2^ dilution series of tetracycline. Cultures were grown for 24 hours at 37°C, shaken at 600 rpm, 3 mm orbital radius, with OD_600_ measured at the end point. Three technical replicates were conducted per clone.

*Growth Curves*

Growth curves were conducted using a Tecan infinite M200 Pro plate reader in 96 well plates. Overnight cultures of clones were sub-cultured to an OD_600_ of 0.5 in fresh NB, and then diluted 1:1000 into 100 μl NB supplemented with 10 μg ml^-1^ TET. Cultures were grown at 37°C with shaking at 300 rpm, 3 mm orbital radius for 24 hours, and OD_600_ was measured every 16 minutes. Three technical replicates were conducted per clone. Growth rates were calculated as the maximum slope of log_2_ transformed OD_600_ covering four time points (~1 hour of growth), lag phase was calculated to end when growth rate reached 10% of the maximum achieved growth rate.

*Statistics*

Statistical analyses were performed in R (version 3.3.3). Comparisons among MIC curves were calculated through comparing the area under the curve, and statistical differences in length of lag phase among clones were calculated using ANOVA, with subsequent Tukey multiple comparison of means. Significant associations between the presence of mutations and level of resistance were calculated for each mutated gene loci using a Pearson Chi-squared test, with the null distribution being approximated by Monte Carlo resampling using 10,000 replicates to account for low expected values using the R package *coin* {coin 1.2-2}[13], followed by Bonferroni correction to correct for multiple testing.

*Data availability*

The sequence data supporting the findings of this study are available at the European Nucleotide Archive, accession: PRJEB28173. All experimental data is available upon request.

**Supplementary Figures**

**Fig. S1** Whole population PCR of *ompF* within populations N1, T4, AT2 and AT5. Short bands of 716 bp indicate on aquisiiton of IS elements within *ompF*. Bands of 1484 bp show the acquisition of IS1, and bands of 1911 bp show the acquisition of IS5 within *ompF*. The insertion of IS elements was never ombserved within the control population evolving with RK2 under no antibiotic selection. Lanes marked with M contain size markers. Three clones were isolated and sequenced from each independent population at transfers 8, 16 and 32.

**Fig. S2** Number of mutations within each sequenced clone from transfers 8, 16, 40 and 80 from populations T4, AT2 and AT5. Solid fill shows chromosomal mutations, dashed fill shows plasmid mutations.

**Fig. S3** The change in tetracycline resistance profile through time in population T4, AT2, and AT5. The vertical line represents the concentration of tetracycline selected for during the evolution experiment. Points represent the collective mean of the three sequenced independent clones from each time point with error bars showing SEM.

**Fig. S4** Growth curves of the ancestral MG1655(RK2) and clones from transfers 8, 16, 40 and sequenced clones from transfer 80 from populations T4, AT2 and AT5 in the presence of tetracycline 10 μg/ml, the same concentration used in the selection experiment. Points are the mean of three replicates with error bars representing SEM.

**Table S2** ANOVA comparison of tetracycline resistance integrals with Tukey multiple comparison tests of **A** population T4, **B** population AT2, and **C** population AT5.

**A** Population T4

| Factor | d.f. | Sum Squares | Mean Squares | F Value | P |
| --- | --- | --- | --- | --- | --- |
| Transfer | 4 | 2.438 | 0.609 | 23.56 | 6.84e-09 |
| Residuals | 30 | 0.776 | 0.026 |  |  |

| Comparison | Difference | P adjusted |
| --- | --- | --- |
| Anc – 8 | -0.075 | 0.916 |
| Anc – 16 | -0.647 | 0.0001 |
| Anc – 40 | -0.464 | 0.0001 |
| Anc – 80 | -0.631 | 0.0001 |
| 8 – 16 | -0.572 | 0.000 |
| 8 – 40 | -0.389 | 0.0001 |
| 80 – 8 | 0.556 | 0.0001 |
| 40 – 16 | -0.183 | 0.138 |
| 80 – 16 | -0.017 | 0.999 |
| 80 – 40 | 0.167 | 0.535 |

**B** Population AT2

| Factor | d.f. | Sum Squares | Mean Squares | F Value | P |
| --- | --- | --- | --- | --- | --- |
| Transfer | 4 | 0.982 | 0.245 | 11.1 | 1.21e-05 |
| Residuals | 30 | 0.663 | 0.022 |  |  |

| Comparison | Difference | P adjusted |
| --- | --- | --- |
| Anc – 8 | 0.033 | 0.994 |
| Anc – 16 | -0.274 | 0.019 |
| Anc – 40 | -0.372 | 0.001 |
| Anc – 80 | -0.179 | 0.482 |
| 8 – 16 | -0.308 | 0.001 |
| 8 – 40 | -0.405 | 0.000 |
| 80 – 8 | 0.211 | 0.231 |
| 40 – 16 | 0.097 | 0.639 |
| 80 – 16 | -0.096 | 0.868 |
| 80 – 40 | -0.193 | 0.315 |

**C** Population AT5

| Factor | d.f. | Sum Squares | Mean Squares | F Value | P |
| --- | --- | --- | --- | --- | --- |
| Transfer | 4 | 1.337 | 0.334 | 7.864 | 0.0002 |
| Residuals | 30 | 1.274 | 0.042 |  |  |

| Comparison | Difference | P adjusted |
| --- | --- | --- |
| Anc – 8 | -0.217 | 0.346 |
| Anc – 16 | -0.379 | 0.020 |
| Anc – 40 | -0.599 | 0.0001 |
| Anc – 80 | -0.380 | 0.111 |
| 8 – 16 | -0.163 | 0.465 |
| 8 – 40 | -0.382 | 0.004 |
| 80 – 8 | 0.164 | 0.756 |
| 40 – 16 | 0.219 | 0.186 |
| 80 – 16 | 0.001 | 1.000 |
| 80 – 40 | -0.218 | 0.515 |

**Table S3** ANOVA comparison of length of lag phase when growing in tetracyclin 10 μg/ml with Tukey multiple comparison tests of **A** population T4, **B** population AT2, and **C** population AT5.

**A** Population T4

| Factor | d.f. | Sum Squares | Mean Squares | F Value | P |
| --- | --- | --- | --- | --- | --- |
| Transfer | 4 | 59.53 | 14.882 | 18.23 | 2.71e-09 |
| Residuals | 50 | 40.81 | 0.816 |  |  |

| Comparison | Difference | P adjusted |
| --- | --- | --- |
| Anc – 8 | 2.179 | 0.0002 |
| Anc – 16 | 3.339 | 0.000 |
| Anc – 40 | 3.392 | 0.000 |
| Anc – 80 | 3.796 | 0.000 |
| 8 – 16 | 1.160 | 0.008 |
| 8 – 40 | 1.213 | 0.005 |
| 80 – 8 | -1.617 | 0.009 |
| 40 – 16 | -0.053 | 0.999 |
| 80 – 16 | -0.457 | 0.863 |
| 80 – 40 | -0.404 | 0.908 |

**B** Population AT2

| Factor | d.f. | Sum Squares | Mean Squares | F Value | P |
| --- | --- | --- | --- | --- | --- |
| Transfer | 4 | 109.33 | 27.331 | 558.7 | <2e-16 |
| Residuals | 50 | 2.45 | 0.049 |  |  |

| Comparison | Difference | P adjusted |
| --- | --- | --- |
| Anc – 8 | -0.246 | 0.214 |
| Anc – 16 | 2.776 | 0.000 |
| Anc – 40 | 2.759 | 0.000 |
| Anc – 80 | 2.583 | 0.000 |
| 8 – 16 | 3.022 | 0.000 |
| 8 – 40 | 3.005 | 0.000 |
| 80 – 8 | -2.829 | 0.000 |
| 40 – 16 | 0.018 | 0.999 |
| 80 – 16 | 0.193 | 0.448 |
| 80 – 40 | 0.176 | 0.543 |

**C** Population AT5

| Factor | d.f. | Sum Squares | Mean Squares | F Value | P |
| --- | --- | --- | --- | --- | --- |
| Transfer | 4 | 110.03 | 27.507 | 25.17 | 1.86e-11 |
| Residuals | 50 | 54.64 | 1.093 |  |  |

| Comparison | Difference | P adjusted |
| --- | --- | --- |
| Anc – 8 | 1.336 | 0.113 |
| Anc – 16 | 3.849 | 0.000 |
| Anc – 40 | 3.726 | 0.000 |
| Anc – 80 | 4.175 | 0.000 |
| 8 – 16 | 2.513 | 0.000 |
| 8 – 40 | 2.390 | 0.000 |
| 80 – 8 | -2.829 | 0.000 |
| 40 – 16 | 0.123 | 0.998 |
| 80 – 16 | -0.316 | 0.977 |
| 80 – 40 | -0.439 | 0.925 |

**Supplementary references**

1. Bottery MJ, Wood AJ, Brockhurst MA. Adaptive modulation of antibiotic resistance through intragenomic coevolution. *Nat Ecol Evol* 2017; **1**: 1364.

2. Harrison E, Guymer D, Spiers AJ, Paterson S, Brockhurst MA. Parallel Compensatory Evolution Stabilizes Plasmids across the Parasitism-Mutualism Continuum. *Curr Biol* 2015; **25**: 2034–2039.

3. Bolger AM, Lohse M, Usadel B. Trimmomatic: a flexible trimmer for Illumina sequence data. *Bioinforma Oxf Engl* 2014; **30**: 2114–2120.

4. Li H, Handsaker B, Wysoker A, Fennell T, Ruan J, Homer N, et al. The Sequence Alignment/Map format and SAMtools. *Bioinformatics* 2009; **25**: 2078–2079.

5. Quinlan AR, Hall IM. BEDTools: a flexible suite of utilities for comparing genomic features. *Bioinformatics* 2010; **26**: 841–842.

6. Li H, Durbin R. Fast and accurate short read alignment with Burrows-Wheeler transform. *Bioinforma Oxf Engl* 2009; **25**: 1754–1760.

7. McKenna A, Hanna M, Banks E, Sivachenko A, Cibulskis K, Kernytsky A, et al. The Genome Analysis Toolkit: A MapReduce framework for analyzing next-generation DNA sequencing data. *Genome Res* 2010; **20**: 1297–1303.

8. Cingolani P, Platts A, Wang LL, Coon M, Nguyen T, Wang L, et al. A program for annotating and predicting the effects of single nucleotide polymorphisms, SnpEff. *Fly (Austin)* 2012; **6**: 80–92.

9. Chen K, Wallis JW, McLellan MD, Larson DE, Kalicki JM, Pohl CS, et al. BreakDancer: an algorithm for high-resolution mapping of genomic structural variation. *Nat Methods* 2009; **6**: 677–681.

10. Robinson JT, Thorvaldsdóttir H, Winckler W, Guttman M, Lander ES, Getz G, et al. Integrative genomics viewer. *Nat Biotechnol* 2011; **29**: 24–26.

11. Tenaillon O, Barrick JE, Ribeck N, Deatherage DE, Blanchard JL, Dasgupta A, et al. Tempo and mode of genome evolution in a 50,000-generation experiment. *Nature* 2016; **536**: 165–170.

12. Paradis E, Claude J, Strimmer K. APE: Analyses of Phylogenetics and Evolution in R language. *Bioinformatics* 2004; **20**: 289–290.

13. Hothorn T, Hornik K, Wiel MA van de, Zeileis A. Implementing a Class of Permutation Tests: The coin Package. *J Stat Softw* 2008; **28**: 1–23.
